# Stretchable conductive elastomer for wireless wearable communication applications

**DOI:** 10.1038/s41598-017-11392-w

**Published:** 2017-09-08

**Authors:** Zhibo Chen, Jingtian Xi, Wei Huang, Matthew M. F. Yuen

**Affiliations:** 1 Department of Mechanical and Aerospace Engineering, The Hong Kong University of Science and Technology, Clear Water Bay, Kowloon, Hong Kong; 2 Hong Kong R&D Centre for Logistics and Supply Chain Management Enabling Technologies Limited, Cyberport Road, Hong Kong

## Abstract

Wearable devices have provided noninvasive and continuous monitoring of physiological parameters in healthcare applications. However, for the comfortable applications of wearable devices on human body, two key requirements are to replace conventional bulky devices into soft and deformable ones and to have wireless wearable communication. In this paper we present a simple, low-cost and highly efficient all-elastomeric conductor that can be used in a soft radio-frequency (RF) transmission line and antenna. We show a stretchable transmission line and two stretchable antennas fabricated with conventional screen printing. The stretchable conductor used in this fabrication method, which is a mixture of Ag and Polydimethylsiloxane (PDMS), can be stretched at high strains while maintaining a high conductivity, low attenuation and feasible radiation performance. The measured conductivity of the stretchable conductor reaches 1000 S/cm. Additionally, the highly conductive printed Ag-PDMS is utilized to construct transmission lines and antennas. The performance of these stretchable components, especially under different conditions of bending, stretching and twisting, are experimentally examined in common wireless-communication frequency bands. Our results demonstrate that printed Ag-PDMS enabled RF passive components have the desired property and quality for wireless wearable communication applications, which would provide new opportunities for wearable healthcare electronics.

## Introduction

Soft and deformable wearable devices have great potential for applications such as on-body medical sensing^1^, body movement detection^2^, electronic skin^3^ and healthcare monitoring^4^. A variety of stretchable wearable components, such as sensor, circuit, battery, etc., have been developed, representing the exciting progress for the next generation of wearable devices for human beings^5–8^.

Wireless wearabilty is a necessary function that helps link the wearable device to the target device, which provides the possible infrastructure for the Body Sensor Network (BSN) or Internet of Things (IoT)^9^. In a wireless communication system, the RF front-end is a key sub-system between the antenna and the digital baseband system. Limited by both developments of material and electronic packaging, the flexible passive RF components that enable the desired RF signal to propagate, such as stretchable antenna^10, 11^ and stretchable transmission line^12^, are now outstanding issues in the design of wireless wearable devices. Finite difference time-domain method (FDTD) and finite element model (FEM) make designing RF passive components easier and characterizing the RF field distribution more precisely. However, the RF passive front-end still faces a great challenge for integration with wearable applications, such as stretchability and flexibility. The soft deformation property results in a more complex antenna design that may sometimes cause failure because of the restrictions of the soft material.

Conventionally, the deposited copper layer used in epoxy based or polytetrafluoroethylene based substrates is a good conductive material for RF transmission and printed antenna applications^13^. However, the rigid conductor and substrate, which can easily crack or fail when subjected to mechanical deformation, such as stretching, twisting or bending, restricts use for wearable applications. Novel soft conductive material, like Nanotube/Nanowire based elastomer, conductive polymer, serpentine metal conductor^14^, etc. has been developed for wearable electronics. Using nanowires as the connecting network in a non-conductive elastomer is an effective way to obtain the high conductivity elastomer even with small stretching^15^. However in RF applications, to maintain the low energy loss of microwave propagation in the conductor, the thickness of the conductor should be higher than the skin depth^16^. A thicker nanowire layer obviously results in higher cost of material preparation and component fabrication. Conductive polymer, such as PEDOT:PSS, is limited by the low conductivity, ultra-thin construction and mechanical instability^17^, and hence is not suitable for wearable RF electronics. Serpentine-based metal conductor^18^ is a promising technology that converts the planar deformation to three-dimensional deformation, avoiding the copper layer cracks. However, the special shape sacrifices the RF performance and also introduces complex radiation effects and electronic coupling.

In this paper we present a simple, low-cost and highly efficient all-elastomeric conductor that can be used in a soft RF transmission line and antenna. We show a stretchable transmission line and two stretchable antennas using conventional screen printing fabrication. The stretchable conductor used in this method, which is a mixture of Ag and PDMS, can be stretched at high strains while maintaining a high conductivity, low attenuation and feasible radiation performance. The measured conductivity from this technique reaches 1000 S/cm. In this report, the highly conductive printed Ag-PDMS is utilized to construct transmission lines and antennas. The performance of these components, especially under different bending, stretching and twisting, are experimentally examined in common wireless-communication frequency bands. The results demonstrate that printed Ag-PDMS enabled RF passive components have the desired property and quality for wireless wearable communication applications.

## Results

### Printed Ag-PDMS preparation and characterization

The preparation of stretchable Ag-PDMS composite is introduced for the stretchable conductor, and the details are included in the method section. The printed pattern and structure were fabricated using a standard stencil printing process. Taking the skin effect of Ag-PDMS conductive elastomer into consideration, the thickness of the stencil mask should be larger than 100 um, and the paste should be viscous enough to resist flowing after printing.

**Figure 1:**
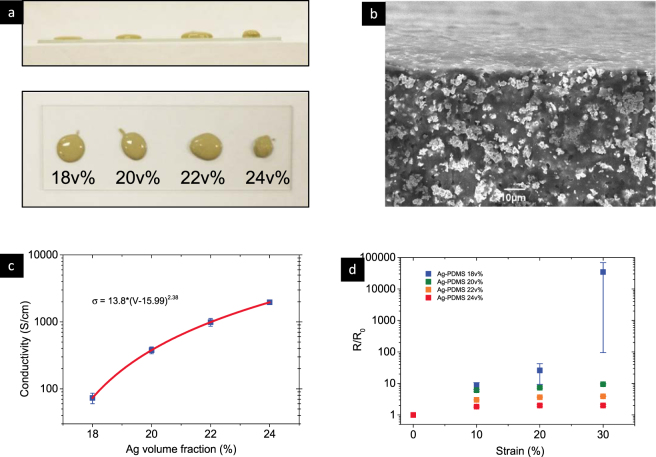
Electro-mechanical properties of different Ag-PDMS composites. (**a**) Ag-PDMS paste with different Ag volume fraction dropped on a glass sheet for 5 minutes; (**b**) cross section of 22 v% Ag-PDMS after curing; (**c**) Conductivity and its percolation simulation with different Ag volume fraction; and (**d**) bulk resistance change under different strain, five samples were made for each resistance measurement.

High resolution patterns could be achieved when using silver volume fraction above 22 v% as the composite would not flow after removing the stencil due to its high viscosity. In Fig. 1a, 18 v% Ag-PDMS sank lower to the surface due to a lower viscosity, almost approaching a liquid state. While 24 v% Ag-PDMS is the most viscous paste in this group and is difficult to spread thinly, being the most viscous paste in this group, would be difficult to spread thinly, making it difficult to be used for printing. The SEM picture in Fig. 1b shows the cross section of the cured 22 v% Ag-PDMS conductive network. It is clearly found that the network was formed by the micro size silver particles, while the surface of the sample is smooth and clean.

### Electro-mechanical properties of Ag-PDMS composite

The mechanical and electrical properties depend on the composite percolation, conductive network and curing process. Conductivity and resistance tensile tests were conducted using printed Ag-PDMS line (50 mm × 2 mm × 0.1 mm) with different Ag volume fraction ranging from 18 v% to 24 v%. The Ag-PDMS paste, after thorough mixing, was printed on a glass slide and cured. After the cured samples were cooled and peeled off, four-probe measurement was used to measure the conductivity while two-probe measurement was used to record the resistance change with strain. The change of viscosity in the Ag-PDMS composite is obvious as the Ag volume fraction increases.

The 18 v% Ag-PDMS paste shows a low electrical conductivity of 70 S/cm and low viscosity, while the 24 v% Ag-PDMS paste shows the opposite properties with high electrical conductivity and gel-like high viscosity. A percolation network model can be used to estimate the electrical conductivity of the Ag-PDMS conductive elastomer. The measured conductivity curve follows the percolation model, indicated by the red line in Fig. 1c. Based on the model, the percolation conductivity threshold is 16 v% with very low conductivity.

Change in the resistance of Ag-PDMS elastomer is caused by the structural deformations, in terms of the effective tunneling distance between the silver particles and the particle density. Therefore, low sensitive relative change of resistance could be achieved with high silver content. The changes in composite resistance R/R0 were applied with 10 mm/min uniaxial strain rate and relax period of 24 hours for each measurement point. As expected, the sample with the lowest silver content showed the highest sensitivity to the strain but with the biggest error due to the unstable conductive network during stretching. More Ag content, such as 22 v% and 24 v% in the PDMS matrix, showed less sensitivity to the resistance changes with elongation and also maintained high conductivity. The more silver content, the more stable the resistance during stretching. However, excessive amount of silver loading increased the composite viscosity and made it difficult to print. To tradeoff between the conductivity, resistance and viscosity, a 22 v% Ag-PDMS paste is used in this paper.

**Figure 2:**
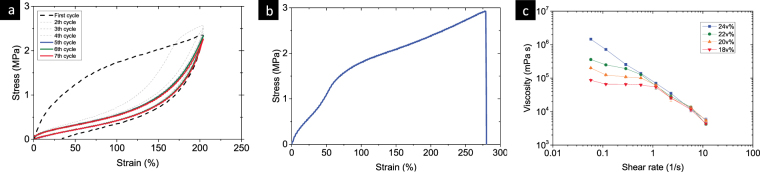
Mechanical properties of the Ag-PDMS composite. (**a**) The stress-strain curve and hysteresis phenomenon and (**b**) maximum rupture strain of 20 v% Ag-PDMS composite; (**c**) The viscosity curves of 18 v%, 20 v%, 22 v%, and 24 v% Ag-PDMS composites.

In the measurements of mechanical properties with around 200% strain amplitude, the stress drops between successive loading cycles. A stationary state with constant stress amplitude and stabilized hysteresis loop is then reached after 5 cycles (Fig. 2a); as shown in Fig. 2b, the elongation of 20 v% Ag-PDMS at break is around 280%, which is close to the reference results^19^. Figure 2c demonstrated the various viscosity curves of different Ag-PDMS composites.

### Printed Ag-PDMS enabled flexible transmission lines

In radio-frequency/microwave circuits, transmission lines are used to guide the propagation of high-frequency signals from one device to another. In contrast to low-frequency signals, high-frequency signals suffer significant losses if the transmission lines are not implemented appropriately. Here, a coplanar-stripline (CPS) transmission line is adopted to investigate the high-frequency properties of Ag-PDMS elastomer. A coplanar stripline consists of two parallel conductive traces in close proximity supported by a substrate. Its main advantage over other transmission-line types is that the mounting of lumped components in shunt or series configuration is much easier.

**Figure 3:**
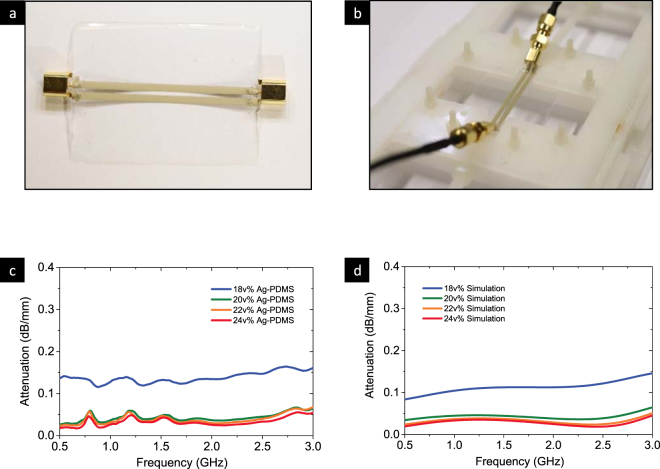
Performance of the transmission lines with different Ag-PDMS composites. (**a**) The outlook of Ag-PDMS transmission line with MCX adapters, (**b**) the tensile test fixture, (**c**) attenuation of the transmission line with different Ag volume fractions, and (**d**) the simulation of attenuation of the transmission line.

For the coplanar stripline implemented in this paper, the substrate is 400 um thick PDMS sheet. The two parallel traces are made in Ag-PDMS elastomer. Their length and width are chosen to be 50 mm and 2 mm respectively. The gap between them is fixed at 1 mm. Once the dimensions of the coplanar stripline are specified, the quality of high-frequency-signal propagation depends on the electrical properties of Ag-PDMS elastomer and PDMS. A coplanar-stripline sample, the test setup and the attenuation results with different silver contents, are shown in Fig. 3. The frequency band studied is up to 3 GHz and covers most wireless applications with wearable devices.

Particularly, a large attenuation change is observed between the 18 v% Ag-PDMS composite and the 20 v% Ag-PDMS composite. In the low Ag volume fraction composite, the Ag particles in the PDMS matrix form a weak conductive network which can be described as closer to the percolation threshold, indicating that the electromagnetic wave cannot propagate through the conductive path smoothly, resulting in the energy loss. On the other hand, the relatively large attenuation in the transmission lines is also a result of the skin depth of the Ag-PDMS composite. All Ag-PDMS transmission lines were printed with a thickness of 120 um, which is about 70% of the skin depth of the 18 v% Ag-PDMS at 1.2 GHz.

### Stretchable antenna for wearable communication system

Wearable antennas are any antenna specifically designed to transmit and receive RF signals while being worn^20^. Considering the attachment between the antenna and human body, there are two main scenarios for the wearable device application; off-body wearing such as a bracelet and handbag, and on-body wearing such as a glove and medical tape. The propagation direction in the off-body scenario points away from the human body, and the matching and the radiation pattern of the respective antennas would change slightly because of the small interaction with the human body^21^. As for the on-body scenario, the electromagnetic interaction with the human body becomes complex due to the biodiversity. The antenna matching, efficiency and radiation pattern sometimes will be quite different from the model and simulation result. In that case, experiments should be planned carefully to determine optimal design.

**Figure 4:**
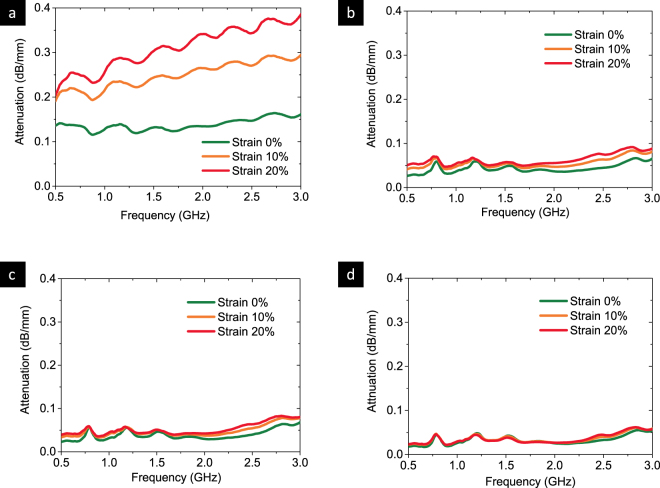
Performance of the transmission line with various stretching. The stretching attenuation in (**a**) 18 v%, (**b**) 20 v%, (**c**) 22 v% and (**d**) 24 v% Ag-PDMS transmission lines.

The stretchable performance of the transmission line is verified with a maximum strain of 20%, as shown in Fig. 4. The change in attenuation of different Ag-PDMS transmission lines under quasi-static uniaxial strain was measured using a tailored polyethylene-only tensile test fixture. The stretching of the Ag-PDMS transmission line does not alter the attenuation much except for the 18 v% Ag-PDMS transmission line, where the average attenuation is above 0.05 dB/mm. As expected, the unstable conductive network in the 18 v% Ag-PDMS transmission line will be further degraded by the stretching, resulting in higher attenuation and conductivity loss.

**Figure 5:**
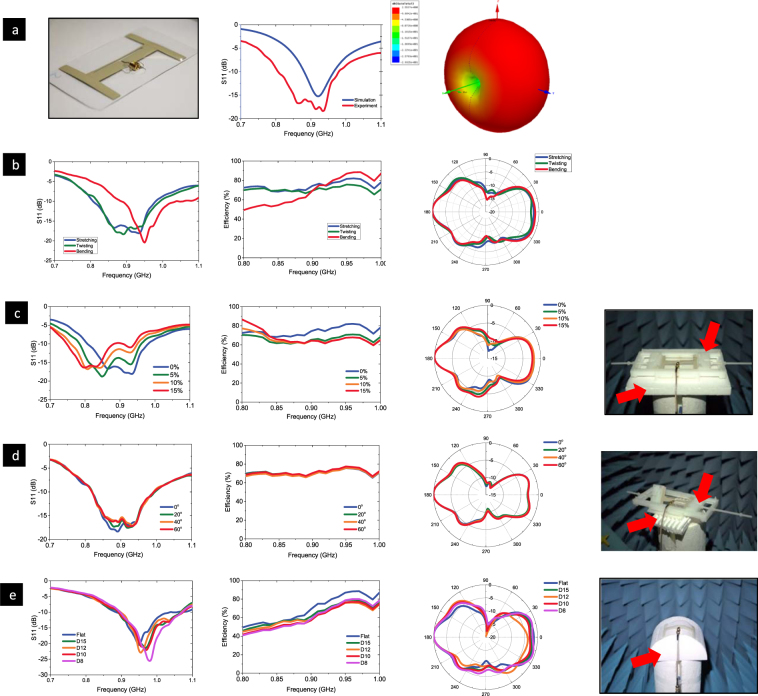
Stretched, Twisted and Bent antenna using 22 v% Ag-PDMS and their transmission performances. (**a**) The outlook of the off-body dipole antenna, the return losses of the unstretched off-body antenna from experiment and simulation, and the 3D radiation pattern of the unstretched off-body antenna, respectively; (**b**) the antenna performance in the initial conditions: 0% stretch, 0-deg twist, and no bending; (**c**) the antenna under stretching; (**d**) the antenna under twisting; and (**e**) the antenna under bending. From left to right in (**b**–**e**) series, are the return loss, radiation efficiency, radiation pattern in the cut-plane pi = 0°, and view of the testing environment, respectively.

For wearable applications, the effective antenna radiation should be maintained to a certain level following the human body deformation. Wearable skin-like antenna is the new demand for wearable communication system. For the first time, the effective radiation of an Ag-PDMS enabled skin-like dipole antenna is experimentally demonstrated in the ultra-high frequency band, at frequencies with 800 MHz to 1000 MHz such as radio frequency identification (RFID), long-term evolution (LTE), global system for mobile communications (GSM) etc. The return losses of the dipole antenna under different stretching, twisting and bending cases were collected with a vector network analyzer, shown in Fig. 5. The antenna was placed on a polyethylene-only fixture for tensile or twist test, and placed on curved foam for the bending test.

There is a clear minimum on the return loss around 920 MHz, as shown in Fig. 5b. Figure 5a shows the center frequency of the dipole antenna. To test the antenna tensile property, a tensile strain ranging from 0% to 15% was applied to the dipole antenna. Figure 5c shows the measured frequency response of the return loss and efficiency. It is found that the radiation efficiency around the initial resonance frequency degrades monotonically as the stretching percentage increases. With the increasing strain, the resonance frequency shifted to a lower frequency due to the increased effective electrical length. The Ag-PDMS conductor can be considered as hyperelastic material, therefore, when the antenna is elongated in the length direction, the width and height shrink proportionally to keep the total volume constant during deformation, resulting in poorer impedance match and a lower radiation efficiency.

As shown in Fig. 5b, dielectrics in the close vicinity of the antenna have a large impact on the antenna performance. In both the stretching and twisting setups, a plastic fixture and white foams are used to support the antenna under test. Therefore, the difference between the 0% stretch case and the 0-deg twist case with respect to the antenna performance is insignificant. However, in the bending setup, without the plastic fixture, only white foams are used. As a result, the no-bend case behaves a bit different. Taking the return loss for instance, it provides a higher resonant frequency and a narrower impedance bandwidth.

According to Fig. 5d, the radiation efficiency does not vary too much as the twisting angle increases. Moreover, the maximum variance is only about 5% between different twisting angles. This indicates that the radiation efficiency is not sensitive to the change of the twisting angle. Meanwhile, the return loss remains stable in most regions regardless of the twisting angle. In Fig. 5e, the resonance frequency shifts to a higher frequency and radiation efficiency degrades as the bending diameter decreases. This is reasonable since bending reduces the electrical length and also introduces more capacitive coupling within the antenna itself. The maximum upward shift in the resonance frequency is about 3.8% (referenced to 950 MHz) during the bending test.

**Figure 6:**
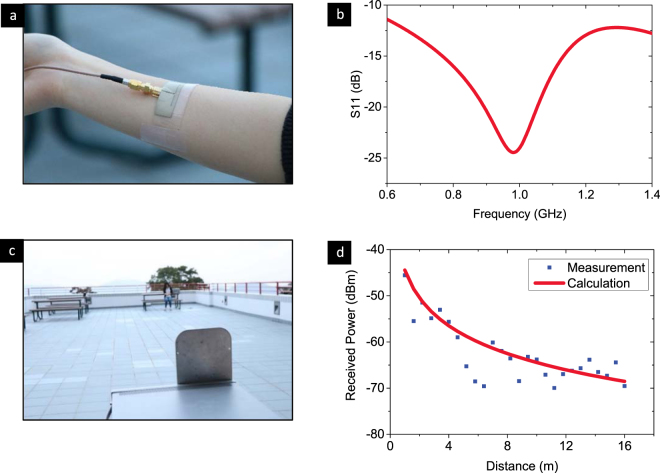
On-body 22 v% Ag-PDMS antenna and its transmission performance in a real application. (**a**) The antenna is directly mounted on a human arm to simulate the final end user case. (**b**) The return loss is simulated for the antenna on a human arm to ascertain its performance in real application conditions. (**c**) The test environment for far field communication showing the relationship between the distance and received power, (**d**) the data are the experimental values of power received by the on-body Ag-PDMS antenna, while the line indicates the expected variation in received power versus distance.

**Figure 7:**
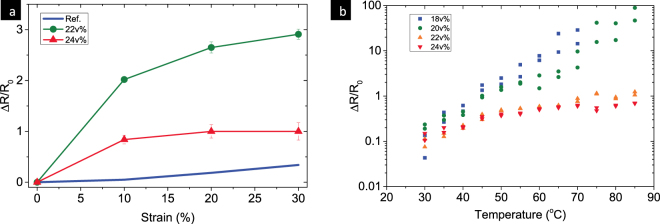
(**a**) A comparison of the change in resistance of Ag-PDMS with that of carbon nanotubes^24^; and (**b**) Temperature effect on the electrical properties change of Ag-PDMS composites.

With the aforementioned demonstration for the soft deformation and RF propagation of the Ag-PDMS based wearable antenna, here we move forward to prove its potential for on-body wireless communication by studying a real life scenario shown in Fig. 6. A modified dipole antenna of around 45 mm × 16 mm was placed on the right arm of a healthy adult male and secured with silicone tape. The testing scenario was on the roof of The Hong Kong University of Science and Technology. The signal generator (Agilent, N5182A) transmitted a 1-GHz RF signal with a right-hand circularly-polarized antenna (6 dBi, Alien Technology) at 0 dbm transmitted power, while the on-body antenna was attached on the human test subject to receive the power using a signal analyzer (Agilent, EXA N9011A). The received power decreased as the testing distance increased in Fig. 6c. There remained a −65 dBm received power at the 10 m distance, indicating a good received-signal strength can be achieved for the application Fig. 7.

## Discussion

We have presented a stretchable Ag-PDMS slot transmission, a stretchable antenna for studying the influence of strain, twist and bending and a stretchable on-body antenna for wearable applications. The feasibility of using conductive elastomer to propagate RF/microwave signals through transmission lines and wirelessly has been demonstrated experimentally.

Encapsulation is a promising solution in wearable applications as it may reduce the EMI, improve the radiation efficiency and ESD protection etc. From the mechanical perspective, the encapsulation can greatly improve the reliability and lifetime. It can distribute the stress concentration to the encapsulation rather than in the device itself, and meanwhile isolate the humidity and oxygen from the antenna.

As shown in Fig. 3, the signal loss is significant at 0.15 dB/mm of conductor, which were due to low conductivity and RF adapters. The 18 v% Ag-PDMS has a poor conductivity (around 70 S/cm) while 22 v% has a conductivity around 1000 S/cm. With a low conductivity like 70 S/cm, the attenuation due to the conductor loss is large. Additional loss results from the RF adapters. The mismatch between the characteristic impedance of the transmission line and that of the RF adapters makes the attenuation due to the RF adapters even larger.

In Fig. 4, frequency range between 500 MHz and 3 GHz was used, which is widely used for wireless communication and covers most commercial radio applications, such as UHF TV channels (500–950 MHz), RFID (860–960 MHz), cellular communication (850–2600 MHz), GPS (1.575 GHz), Bluetooth (2.4–2.485 GHz), Wifi (2.4–2.5 GHz) etc.

Our wearable device is designed to mount on the skin (excluding the joint areas). The stretchable property of Ag-PDMS composite is essential, since the sensing precision, repeatability, stability and adhesion to skin are all determined by the sensors’ capability of following the skin motion, which causes skin deformation up to 20%^22^.

Our data suggested that the stretchable Ag-PDMS elastomer can tolerate deformation, especially in the stretching status, and resist the signal loss of RF passive front-end for wearable applications.

## Methods

### Fabrication of the Ag-PDMS composites

The PDMS composite was prepared by mixing with 10:1 weight ratio of base and cross linker (Sylgard 184 silicone kit, Dow Corning) and mixed in a planetary mixer (Thinky ARE-250) for 1 minute at 2000 rpm. After that, silver powder (2–3.5 micron, Sigma Aldrich) was dispersed by pre-mix for 30 seconds at 500 rpm, and full mixing for 10 minutes at 2000 rpm and degassing for 30 seconds at 2000 rpm. The viscous paste obtained was stored in a freezer at −24 °C for 30 minutes and then thawed. Prior to usage, the Ag-PDMS paste was mixed for 5 minutes at 2000 rpm and degassed for 30 seconds at 2000 rpm. The mechanical properties of PDMS composites were measured by a Universal Testing Machine. The viscosity of PDMS composites was measured by a coaxial cylinder rotational viscometer. Five samples were made for each resistance measurements.

### Transmission line and antenna design

The transmission line was designed with the dimension of 2 mm width × 50 mm Length × 120 um thickness with symmetry lines, under the PDMS substrate of 50 mm length × 30 mm width × 400 um thickness. The antennas were designed with a T-match-dipole antenna of specific dimension see reference^23^.
